# Self-Sensing Soft Skin Based on Piezoelectric Nanofibers

**DOI:** 10.3390/polym15020280

**Published:** 2023-01-05

**Authors:** Giacomo Selleri, Francesco Mongioì, Emanuele Maccaferri, Riccardo D’Anniballe, Laura Mazzocchetti, Raffaella Carloni, Davide Fabiani, Andrea Zucchelli, Tommaso Maria Brugo

**Affiliations:** 1 Department of Electrical, Electronic, and Information Engineering, University of Bologna, Viale Risorgimento 2, 40136 Bologna, Italy; 2 Department of Industrial Engineering, University of Bologna, Viale Risorgimento 2, 40136 Bologna, Italy; 3 Department of Industrial Chemistry “Toso Montanari”, University of Bologna, Viale Risorgimento 4, 40136 Bologna, Italy; 4 Faculty of Science and Engineering—Bernoulli Institute for Mathematics, Computer Science and Artificial Intelligence, University of Groningen, Nijenborgh 9, 9747 AG Groningen, The Netherlands

**Keywords:** e-skin, piezoelectric, nanofibers, self-sensing, electrospinning, PVDF-TrFE

## Abstract

The development of electronic skins and wearable devices is rapidly growing due to their broad application fields, such as for biomedical, health monitoring, or robotic purposes. In particular, tactile sensors based on piezoelectric polymers, which feature self-powering capability, have been widely used thanks to their flexibility and light weight. Among these, poly(vinylidenefluoride-trifluoroethylene) (PVDF-TrFE) presents enhanced piezoelectric properties, especially if manufactured in a nanofiber shape. In this work, the enhanced piezoelectric performances of PVDF-TrFE nanofibers were exploited to manufacture a flexible sensor which can be used for wearable applications or e-skin. The piezoelectric signal was collected by carbon black (CB)-based electrodes, which were added to the active layer in a sandwich-like structure. The sensor was electromechanically characterized in a frequency range between 0.25 Hz and 20 Hz—which is consistent with human activities (i.e., gait cycle or accidental bumps)—showing a sensitivity of up to 4 mV/N. The parameters of the signal acquisition circuit were tuned to enable the sensor to work at the required frequency. The proposed electrical model of the nanofibrous piezoelectric sensor was validated by the experimental results. The sensitivity of the sensor remained above 77.5% of its original value after 10^6^ cycles of fatigue testing with a 1 kN compressive force.

## 1. Introduction

With the rapid development of the robotic field and artificial intelligence systems, the design of sensors which can acquire information from the surrounding environment is becoming an urgent topic. In recent times, the growing demand for wearable electronics has rapidly accelerated the transition from traditional, rigid microelectronics towards lightweight and flexible devices. Beyond their flexibility, these devices are required to be highly sensitive to a variety of parameters, such as temperature, humidity, strain and pressure. In the case of pressure monitoring, such sensors have to be designed according to their final application. For instance, flexible and stretchable electronics have been developed to measure various biological signals and have given rise to the so-called electronic skin [1], which can be installed in robotic systems or encapsulated in wearable materials. In the medical field, e-skins have been used to monitor health parameters, such as blood pressure, muscle movements, pulses, etc. [2], or—in engineering structures—to manufacture smart composite materials by integrating them onto the composite surface and creating a network of receptors. A variety of flexible pressure sensors have been proposed in different works, based on different working principles. Among these, pressure sensors based on piezoresistive effects convert the mechanical deformation of the material into a variation of electrical resistance and are able to detect a wide range of mechanical loads [3]. The sensitivity of piezoresistive sensors strongly depends on the flexibility and the electrical conductivity of the materials they are composed of. Consequently, the addition of conductive elements—such as carbon black (CB) [4], carbon nano-tubes (CNT) [5], and graphene—to elastic matrices represents a promising strategy to enhance the sensitivity, as does the use of conductive polymers (i.e., PEDOT:PSS [6]). Capacitive-based flexible pressure sensors are a deeply investigated strand among different works in the literature. Their working principle is based on the variation of the capacitance as a response to mechanical stimuli. As for piezoresistive sensors, the sensitivity of capacitive-based sensors is strictly correlated to the elasticity of the dielectric film. Polydimethylsiloxane (PDMS) is widely used for this kind of sensor because of its Young’s modulus, transparency and compliance with human tissue [7,8,9,10]. Efforts have been made to enhance the sensitivity of capacitive-type pressure sensors by filling a dielectric elastomer with conductive CB nanoparticles [11] or multiwalled carbon nanotubes (MWCNTs) [12]. Improved sensitivities of capacitive sensors have been achieved with micro- or nanostructures in the dielectric layer—such as pores, microspheres and microcylinders—to increase the contact area and the sensitivity in a specific load direction. Niu et al. fabricated a film of poly(vinylidenefluoride-co-trifluoroethylene) with interlocked asymmetric nanocones, which showed enhanced sensitivity as a result of the amplified variation of the dielectric constant and thickness of the film in the case of vertical stresses [13]. However, despite their high sensitivity and the ultra-stretchable configurations explored in recent times, capacitive- and resistive-based pressure sensors still rely on an external power supply to function. In this context, the exploitation of the triboelectric and piezoelectric effects is a promising strategy to develop self-powered flexible tactile sensors. A triboelectric generator is based on the induction of a potential difference at the interface between two materials with opposite polarities [14]. When a mechanical compression is applied to the bi-layered system, the friction at the interface generates electrostatic charges which can be transferred to an external circuit, thus converting the mechanical energy into electrical energy or signals. Because triboelectric sensors are characterized by a simple and versatile design and can achieve high-output performances, they have attracted enormous interest in the last few years for the development of e-skins or tactile wearable sensors [15,16]. On the other hand, the self-powering capability of piezoelectric materials stems from the intrinsic deformation of the crystal lattice of the piezo-material as pressure is applied to it. Consequently, a charge distribution occurs on the two opposite surfaces of the material, and an electric signal proportional to the applied pressure can be measured.

Among the piezoelectric materials, the piezo-polymers are becoming highly attractive in many fields, such as SHM systems and robotic systems. The advantageous features of polymers such as PVDF (and its co-polymers) include its variety of sizes and shapes (e.g., films, nanofibers, etc.), its flexibility and its potential to be embedded in very remote locations [17,18]. Various studies have been dedicated to the development of PVDF-based piezoelectric pressure sensors and electronic skin to be used for wearable devices, (i.e., for human motion detection [19]). Among these, the development of flexible sensors based on PVDF nanofibers—produced via electrospinning—has been widely explored for different reasons [20]. First, studies have shown that electrospinning favors the formation in the polymer of the β phase, the one responsible for the piezoelectric behavior [21], thus leading to highly sensitive piezoelectric membranes. For example, Maity et al. introduced an e-skin metal-free based method to fabricate a piezoelectric sensor where highly aligned PVDF nanofibers (NFs) arrays were used as the piezoelectric active mat, and conductive polyaniline coated PVDF (PANI–PVDF) NFs were used as a flexible electrode [22,23]. Moreover, because the nanofibrous mats present a high porosity grade, their integration with hosting matrices—thermoset or thermoplastic—allows for the fabrication of self-sensing layers which are made of the required material, according to the final application. For instance, highly sensitive PVDF nanofibrous mats were embedded in polydimethylsiloxane (PDMS) (up to 254 mV/N), and flexible electrodes were manufactured by using flexible polymeric conductive electrodes [24]. This aspect is a key feature in the case of manufacturing electronic skins to be bonded onto composite material surfaces, such as robotic hands or prosthetic soles [25]. The use of traditional PVDF films would imply issues regarding the interface adhesion between the fluorinated polymer and the hosting material, due to chemical incompatibility reasons. Instead, the intimate contact between nanofibers and the embedding erases adhesion issues, directly creating a smart layer.

Herein, a tactile sensor based on piezoelectric nanofibers of PVDF-TrFE is proposed. The nanofibrous membrane was produced via electrospinning and embedded in a polymeric matrix to manufacture a flexible piezoelectric sensor. The hosting matrix was designed with the purpose of fabricating an e-skin which can be used for wearable devices or be bonded onto curvilinear surfaces of a composite material, such as the steering wheel of a car or a robotic limb. With the aim to preserve the elasticity of the matrix and avoid traditional metallic electrodes, a stretchable electrode was manufactured by dispersing conductive carbon black (CB) nanoparticles in the same matrix used for the nanofiber embedding. A wide characterization of the piezoelectric signal was then performed in terms of the operating frequency range and compressive force limits. In particular, with the final purpose of using the piezo-sensor as an e-skin or wearable device, the investigations were performed in the low-frequency region (from 0.25 to 20 Hz).

## 2. Materials and Methods

### 2.1. Electrospinning Process

The polymeric solution was prepared by dissolving of the piezoelectric copolymer (PVdF-TrFE 80/20 mol%, Curie temperature Tc = 133 °C, kindly provided by Solvay S.p.A. Milan) in dimethylformamide (DMF) and acetone (AC), with the following weight percentages (%wt): 7% PVDF-TrFE, 23% DMF and 70% AC. The solution was magnetically stirred for 2 h at 50 °C to facilitate the polymer solubilization. 

The electrospinning apparatus (Spinbow Lab Unit, Spinbow S.r.l., San Giorgio di Piano, Italy) consists of a metallic needle connected to the high voltage generator (set at 15 kV) and a low-speed rotating drum collector connected to the ground. The polymeric solution was pumped into a syringe with a flow rate set at 0.8 mL/h and the distance between the high voltage needle and the drum collector was set at 15 cm. The electrospinning process took place for 3 h.

### 2.2. Polarization Process

**Figure 1:**
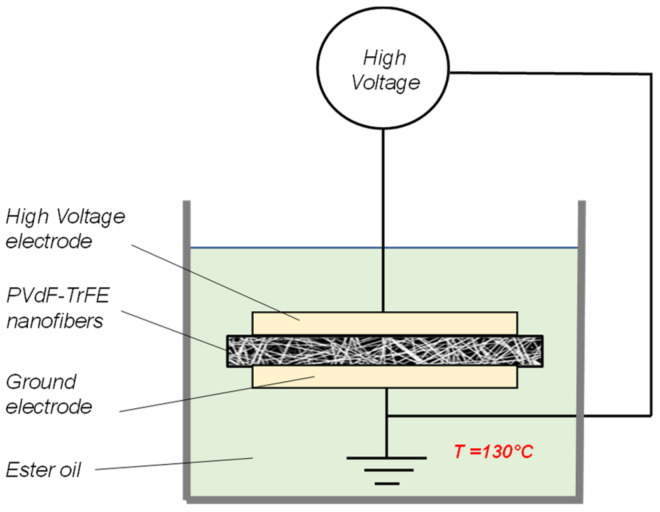
Schematic representation of the polarization setup.

The macroscale piezoelectric behavior of a PVDF-TrFE-based sensor or nanogenerator is strictly correlated to the dipole orientation of the polymeric chains. In polymeric thin films the dipole orientation is typically induced by applying a strong external electric field [26], which forces the ferroelectric domains to align in the electric field direction. The use of high temperatures (close to the Curie temperature T_c_) during the polarization process facilitates the dipole movements. In the case of nanofibrous membranes, during the electrospinning process the evidence of the preferential orientation of CF_2_ dipoles in the PVDF-TrFE nanofiber was proven [27], and a piezoelectric response of the nanofibers was measured after electrospinning. However, a subsequent poling process is still required to enhance the piezo-output of the nanofiber mat. Differently from a PVDF-TrFE thick film, the application of an external electric field could easily result in electrical discharges even for low values of the electric field due to the high porosity grade of nanofibrous mat. Therefore the polarization process was carried out in an ester oil bath (FR3 natural ester, Cargill, electrical breakdown = 70 kV/mm), as schematically represented in Figure 1. The ester oil penetrates the air pores of the nanofibrous membrane and allows a substantial increase in the electric field value applied between the high-voltage and ground electrodes. The electric properties (dielectric constant and electrical conductivity) of the used FR3 natural ester were taken into account to evaluate the electric field distribution between the two phases (oil and nanofibers) of the system, as described in detail in [28]. The applied electric field was set at 20 kV/mm, and the process took place for 10 min at 130 °C. Afterwards, the temperature was decreased to room temperature while keeping the electric field on. In the end, the oil was fully removed from the pores of the membrane by soaking it in a cyclohexane bath for 1 h without stirring to avoid any damaging of the nanofiber membrane.

### 2.3. Nanofiber Integration

Once the PVDF-TrFE nanofibers were poled and a macroscale piezoelectric behavior was conferred to the layer, the nanofibrous mat was embedded in a hosting material in order to manufacture the active layer of the flexible piezoelectric sensor. In particular, with the aim of producing a soft piezoelectric skin, the embedding medium was a mixture of blocked isocyanate polyurethane (PU) prepolymer (Synthane 2095, Synthesia Technology, Barcelona, Spain) and epoxy resin (Itapox 108, kindly provided by Ddchem S.l.r., Verona, Italy). The use of such a mixture conjugates the flexibility of such a material with the possibility of bonding it to other composite material surfaces. A curing agent (Itamine CA119, Ddchem S.l.r., Verona, Italy) was added after the stirring of the two components. The weight percentages (%wt) of the produced blend are as follows: 39% epoxy resin, 39% polyurethane and 22% curing agent. The embedding medium was poured onto the nanofibrous membrane and penetrated the air pores. The excess material was removed by means of a blade, and the curing process was carried out for 2 h at 50 °C and atmospheric pressure.

### 2.4. Carbon Black-Based Electrodes

In order to develop the entire structure of the sensing skin and to collect the piezoelectric signal, electrodes have to be placed on the two opposite surfaces of the active layer. Electrodes such as aluminum foils or gold-sputtered layers are widely used for this purpose, but in the case of flexible devices the foils could rip or crumple, and the thin metallization could present some discontinuities. Moreover, the adhesion between the metallic layer and the polymeric matrix could worsen in case of bending or strong mechanical impacts. To overcome these drawbacks, in this study the electrodes were fabricated by dispersing carbon black (CB) conductive nanoparticles (Printex XE2B, BET surface area = 1000 m^2^/g, average particle size = 30 nm) in the same blend of epoxy resin and polyurethane used for the nanofiber integration in Section 2.3.

**Figure 2:**
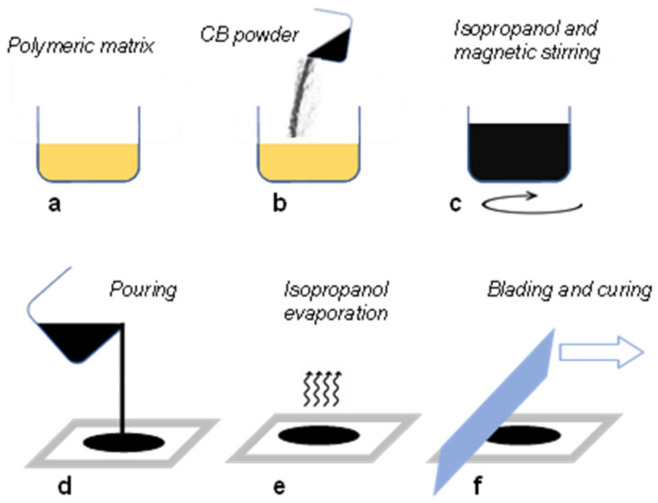
CB-based electrode manufacturing: (**a**) epoxy-polyurethane mixing; (**b**) CB powder addition; (**c**) CB dispersion by magnetic stirring with isopropanol; (**d**) pouring on Teflon substrate; (**e**) isopropanol solvent evaporation; (**f**) blading for film thickness calibration and final curing.

**Figure 3:**
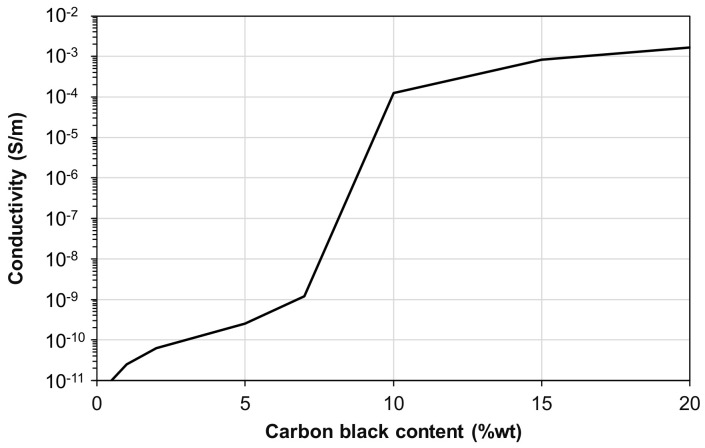
Electrical conductivity of the semi-conductive layer as a function of the CB content, adapted with permission from Ref. [25] 2022. Selleri et al.

The carbon black nanoparticles were dispersed by magnetically stirring the liquid formulation without the curing agent (epoxy resin and PU). The addition of 300%wt of isopropanol facilitated a homogeneous dispersion of the CB nanoparticles in the mixture, as shown in Figure 2a–c. After 120 h, the curing agent was added and mixed for another 5 min, and the liquid solution was placed on a Teflon support with a pouring mask (Figure 2d). A heating process of 30 min at 40 °C was needed in order to facilitate the isopropanol’s evaporation and avoid air bubbles forming inside the layer during the curing process (Figure 2e). In the end, as represented in Figure 2f, a uniform, isopropanol-free, thin layer of 100 ± 8 µm was obtained by means of a blade, and the curing process was carried out. As previously observed in [25], by varying the amount from 1%wt to 20%wt, the electric conductivity suddenly increases (percolation threshold) in correspondence with a CB content equal to 10%wt (Figure 3). However, a further increase of the CB nanoparticles above 20%wt induced a fragile behavior of the layer.

**Figure 4:**
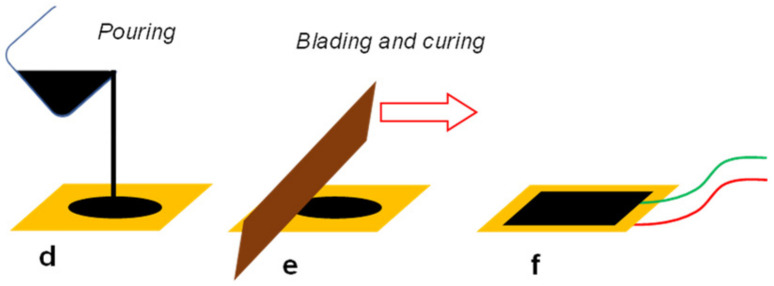
CB electrode deposition and curing with signal cables.

Therefore, as the best trade-off between electrical conductivity and mechanical performance, a carbon black content of 10%wt was selected for the final manufacturing of the electrodes. The electrodes were co-cured directly on the two opposite surfaces of the piezoelectric layer of Section 2.3 according to the abovementioned procedure, as represented in Figure 4. In the end, the same process was repeated to add shield electrodes to the sensor. The functionality of the external electrodes is to remove external noise and triboelectric effects which affect the piezoelectric response [29]. During the curing process of each semiconductive layer, cables (430-FST, Micro-Measurements, Raleigh, NC, USA) coated with a Teflon jacket were placed within the electrode layers.

### 2.5. Characterization Techniques

The morphology of the piezoelectric nanofibers and the final structure of the piezoelectric sensor were investigated via micrograph analyses by using a Phenom Pro X scanning electron microscope (SEM). The nanofibrous membrane was observed after electrospinning, and the average diameter was determined from a sample of 100 nanofibers by using an image analysis software (ImageJ). The multiple-layer structure of the sensor was observed in a cross section after a fragile cut in a nitrogen bath. The samples were sputter-coated with gold before the evaluation.

The piezoelectric strain coefficient *d*_33_ of the nanofibrous membrane was measured by means of a piezometer (*d*_33_ PiezoMeter System, Piezotest, Singapore, www.piezotest.com, accessed on 15 November 2022), which stresses the samples with a compressive sinusoidal force oscillating between 0.25 and 0.5 N at 100 Hz. The *d*_33_—defined as the ratio between the generated charges and the applied force—was measured also on the piezoelectric active layer after the nanofibers’ integration into the hosting structure (Section 2.3).

**Figure 5:**
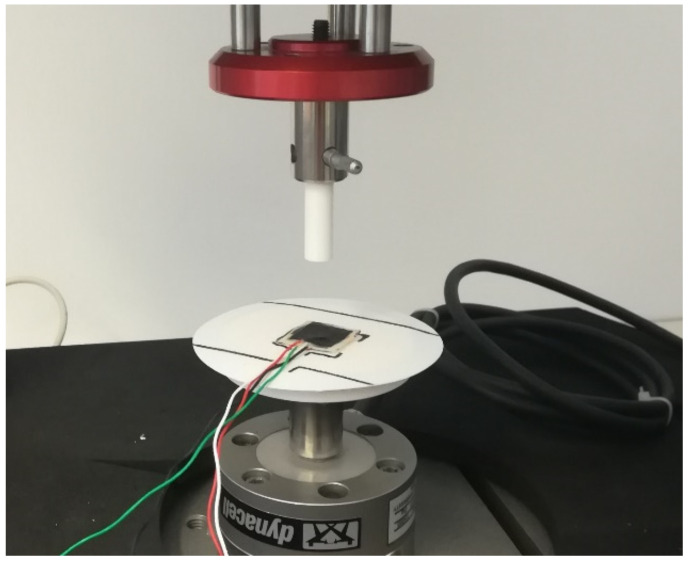
Compression force test setup.

The piezoelectric sensitivity of the flexible sensor was studied for a frequency range between 0.25 Hz and 20 Hz by using an ElectroPuls E1000 tensile machine (Instron, Norwood (MA), USA) equipped with a 2 kN load cell. The sensor was compressed between a flat plate and a cylindrical indenter with a diameter of 1 cm, as shown in Figure 5. The piezoelectric output was acquired by a high-impedance input amplifier (AD795JRZ), and the shield electrodes were connected to the ground. The amplifier was set to the buffer mode, so no signal amplification was performed. Piezoelectric and load cell signals were synchronously acquired for different values of shunt resistances and capacitances, which were connected in parallel to the piezoelectric input.

**Figure 6:**
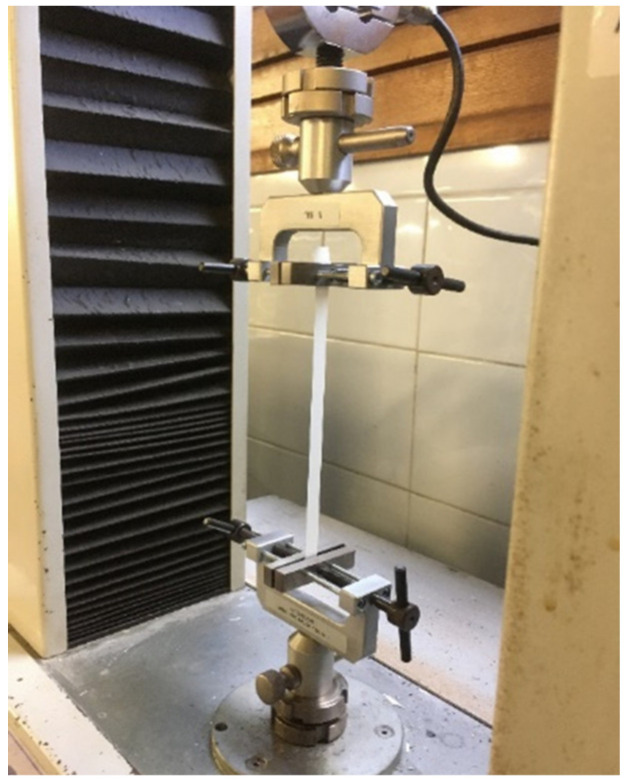
Tensile test setup.

To evaluate the modulus of elasticity of the polymer matrix described in Section 2.3, a tensile test was carried out on a specimen with 80 × 10 × 0.15 mm dimensions. The test was performed by using a TC10 testing machine at a strain rate of 10 mm/min, and the force was measured by 100 N load cell, as shown in Figure 6.

## 3. Results

The micrograph analyses of the electrospun nanofibers and the structure of the piezoelectric sensor are reported in Section 3.1. In Section 3.2 the proposed piezoelectric model is illustrated, and the experimental piezoelectric response is reported in terms of force-detecting capability, response over a frequency range and linearity from Section 3.2 to Section 3.4. In the end, accelerated fatigue tests on the sensor were performed as described in Section 3.5, and the elasticity of the sensor was measured (Section 3.6).

### 3.1. Micrograph Analyses

**Figure 7:**
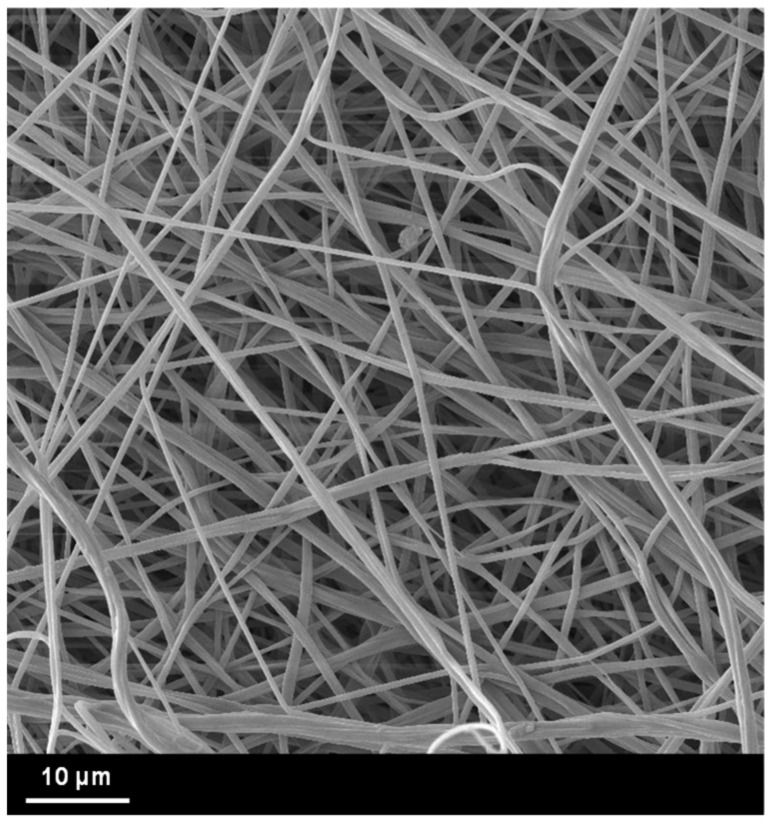
SEM image of the PVDF-TrFE nanofibers.

The electrospun PVDF-TrFE nanofibers showed a randomly oriented disposition and a bead-free morphology (see Figure 7). The diameter distribution of the nanofibers was measured to be 380 ± 92 nm, and the total thickness of the membrane was 107 ± 6 µm.

### 3.2. Force Detecting

**Figure 8:**
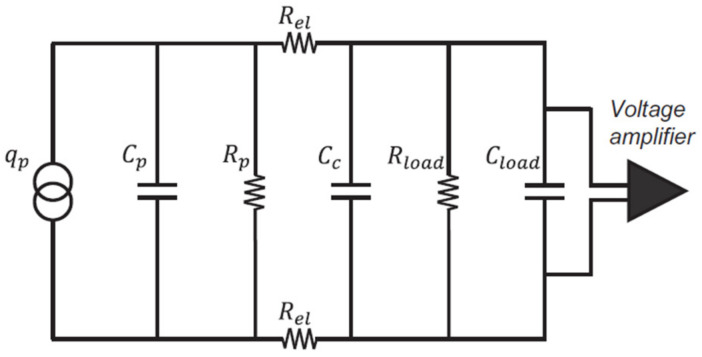
Equivalent electric circuit of the piezoelectric sensor connected to a voltage amplifier.

According to [25], a piezoelectric element can be modeled as a charge generator $q_{p}$, whose equivalent electric circuit is represented in Figure 8. The equivalent capacitance and resistance ($C_{p}$ and $R_{p}$, respectively) of the piezoelectric layer are connected in parallel to $q_{p}$ and $C_{c}$, representing the capacitance associated with the signal cables (55 pF). The resistances of the semiconductive CB-based electrodes $R_{el}$ are connected in series to $q_{p}$ with a value equal to 4 kΩ. The shunt resistance $R_{load}$ and the capacitance $C_{load}$ were connected in parallel to $q_{p}$ for the signal conditioning of the piezoelectric output voltage. Indeed, by varying the values of $R_{load}$ and $C_{load}$, the $RC_{\;}$ constant of the circuit is varied, and the output voltage of the sensor can be evaluated by applying Kirchhoff’s laws to the equivalent circuit. In the case of a sinusoidal load $F\left(t\right)=F*\sin\left(\omega t\right)$, the piezoelectric output voltage can be written as:

(1) Vt=FωRd33ω2R2C2+1e−tRC+cosωt+ωRCsinωt

where ω=2π/T, T is the period, $R=2*R_{el}+\left(R_{load}*R_{p}\right)/(R_{load}+R_{p})$, and $C=C_{p}+C_{c}+C_{load}$. A detailed analytical demonstration can be found in [29].

Before polarization, the piezoelectric strain coefficient *d*_33_ of the electrospun nanofibrous membrane presented a null value. However, after the polarization process, the *d*_33_ increased to 14 pC/N, thus confirming the effectiveness of the proposed method. Furthermore, the *d*_33_ was measured after the nanofibrous membrane was integrated into the mixture of epoxy resin and polyurethane, as described in Section 2.3. As predicted, the *d*_33_ coefficient of the composite layer decreased to 0.54 pC/N, as the hosting mixture reduced the stress transferred to the nanofibers and, consequently, the generated charges.

**Figure 9:**
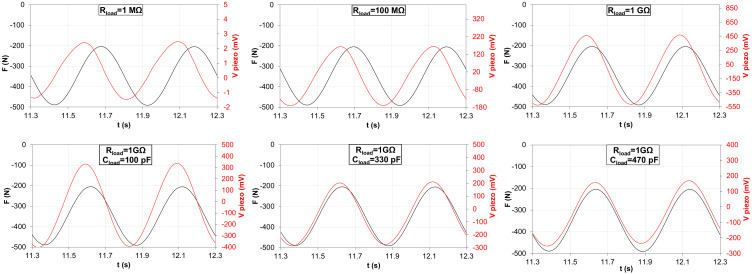
Piezoelectric output voltage vs. compressive sinusoidal force for different values of $R_{load}$ and $C_{load}$.

The variation of the *RC* constant of the equivalent circuit of Figure 8 results in a variation of the amplitude of the piezoelectric response and a phase shift between the piezoelectric curve and the measured force [29]. As an example, in Figure 9 the piezoelectric output of the flexible sensor, generated as response of a 200–500 N peak-to-peak compressive sinusoidal load at a frequency of 2 Hz, is reported for different *RC* values of the equivalent circuit by changing the values of $R_{load}$ and $C_{load}$.

As is observable in the graphs of Figure 9, a remarkable phase shift between the piezoelectric output voltage (orange curve) and the load cell signal (blue curve) occurs in case of low *RC* values, as shown in the case of $R_{load}$ = 1 MΩ. The phase shift between the two signals is gradually reduced by increasing the value of $R_{load}$, whose maximum value in this work was set at 1 GΩ. In this case ($R_{load}$ = 1 GΩ), the amplitude of the piezoelectric response presents its maximum value, but still, a phase shift within the applied force curve is noticeable. Consequently, a strategy to boost the *RC* constant of the circuit consisted of adding a capacitor $C_{load}$ in parallel to $R_{load}$, which was fixed equal to 1 GΩ. The $C_{load}$ capacity values tested in this work are equal to 100 pF, 330 pF and 470 pF. As is observable in Figure 9, the higher the $C_{load}$ value, the lower the phase shift between the two curves, in particular in the case of $C_{load}$ = 470 pF, where the piezoelectric output curve and the load cell signal basically overlap.

In conclusion, the obtained results clearly illustrate the effect of the variation of the *RC* value on the piezoelectric output signal, which is affected both in terms of phase shift and amplitude. The peak-to-peak voltage output has its minimum value equal to 4 mV for $R_{load}$ = 1 MΩ, and it reaches is maximum for $R_{load}$ = 1 GΩ (V_piezo_ = 1.03 V). The further addition of $C_{load}$ is a compromise between a lowering of the amplitude and the removal of the phase shift.

### 3.3. Sensitivity versus Frequency

The piezoelectric equivalent circuit of Figure 8 behaves as a high-pass filter, whose cutoff frequency ($f_{c}$) depends on the capacitance C and the resistance R and can be easily calculated as:

(2) $$f_{c}=\frac{1}{2\pi RC}$$

Depending on the designed application of the sensor, the signal-conditioning circuit parameters ($R_{load}$ and $C_{load}$) have to be tailored to obtain a constant sensitivity (mV/N) vs. force frequency (Hz) in the operational range, where the sensitivity is expressed as the ratio between the peak-to-peak output voltage and the applied force. Therefore, an experimental campaign was conducted with the aim of investigating the cutoff frequency variation as a function of $R_{load}$ and $C_{load}$. For each curve, the cutoff frequency $f_{c}$ corresponds to a sensitivity attenuation equal to 3 dB (equal to 1/√2) of the Bode diagram.

**Figure 10:**
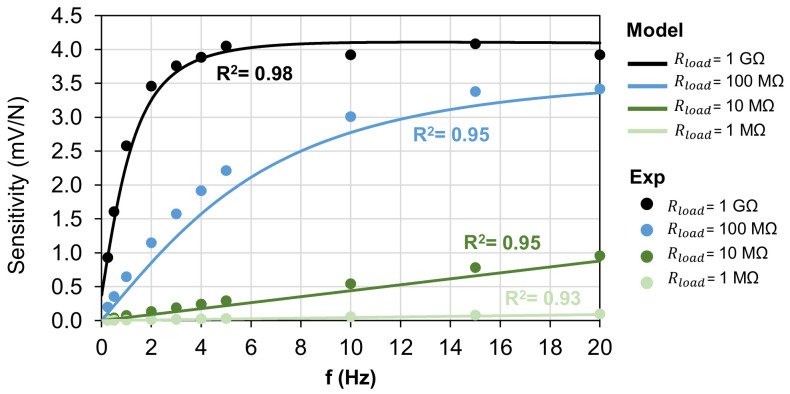
Sensitivity of the piezoelectric sensor vs. the frequency of the compressive force for different values of $R_{load}$.

First, the experimental tests were performed without connecting the capacitance $C_{load}$ to the circuit and just varying the $R_{load}$ value (1 MΩ, 10 MΩ, 100 MΩ and 1 GΩ), as reported in the graph of Figure 10, where the dots are the measured sensitivities and the continuous lines are the sensitivities of the theoretical model, calculated according to Equation (1). As is observable, the sensitivity increases with the $R_{load}$ value, whereas the cutoff frequency decreases down to 1.7 Hz in the case of $R_{load}$ = 1 GΩ.

**Figure 11:**
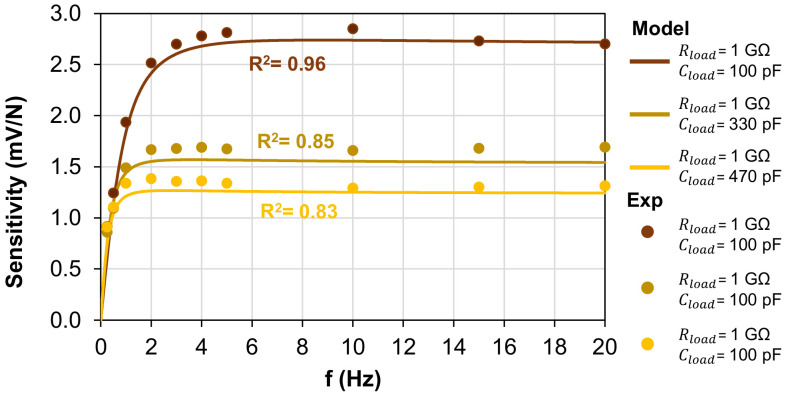
Sensitivity of the piezoelectric sensor vs. frequency of the compressive force for $R_{load}$ = 1 GΩ and different values of $C_{load}$.

Standard human activities can typically vary in a frequency range from 1 Hz, such as the gait cycle, to 10 Hz in case of impacts with objects or surfaces. Therefore the cutoff frequency of the system should be well below the 1.7 Hz value found above. Hence, the addition of the capacitance $C_{load}$ in parallel to the piezoelectric output is investigated to lower $f_{c}$ and to make the sensor suitable for a wider application field. As plotted in Figure 11, by fixing $R_{load}$ = 1 GΩ and adding $C_{load}$, the cutoff frequency was decreased to 0.5 Hz for $C_{load}$ = 470 pF. In this case, the amplitude of the piezoelectric voltage was decreased to 1.3 mV/N, which was consistent with Equation (1).

### 3.4. Linearity

**Figure 12:**
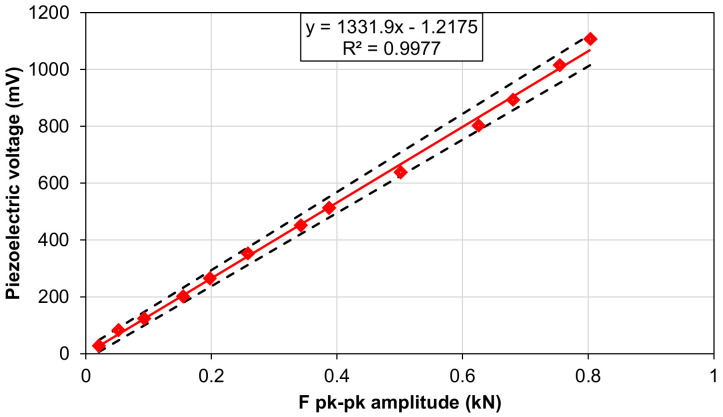
Output voltage of the piezoelectric sensor for increasing compressive forces, with $C_{load}$ = 470 pF and $R_{load}$ = 1 GΩ.

For the aforementioned configuration ($C_{load}$ = 470 pF and $R_{load}$ = 1 GΩ), a linearity analysis has been performed on the piezoelectric sensor. The piezoelectric peak-to-peak output voltage was measured for different exciting forces (Figure 12). The frequency was set at 2 Hz, and the sample was stressed by a compressive sinusoidal load with different amplitudes and a lower peak of 200 N. The piezoelectric sensor shows a sensitivity equal to 1332 ± 62 mV/kN with a coefficient of determination R^2^ equal to 0.997.

### 3.5. Fatigue Behavior

**Figure 13:**
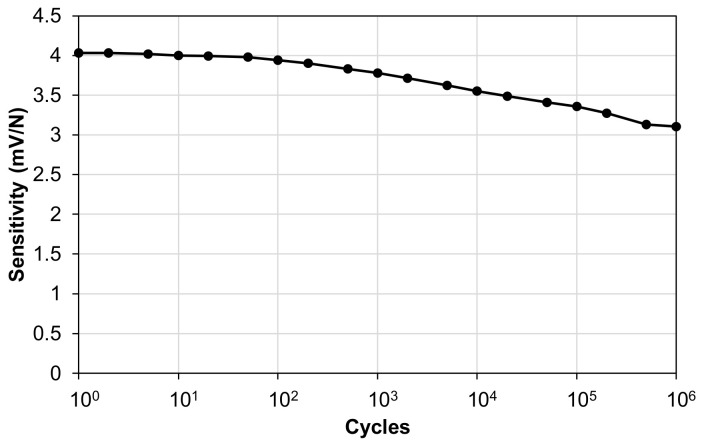
Piezoelectric sensor fatigue test.

The sensitivity of the piezoelectric sensor was measured during accelerated cyclic fatigue tests by applying a sinusoidal load oscillating between 400 N and 1000 N in compression at a frequency of 10 Hz for 10^6^ cycles. The force range was chosen to simulate typical gait cycle ground reaction forces. $R_{load}$ was set at 1 GΩ, and $C_{load}$ = 0, which is the condition with the highest amplitude of piezoelectric response. As is observable from the graph in Figure 13, at the end of the 10^6^ cycles of fatigue testing, the sensitivity of the piezoelectric sensor decreased from 4 mV/N to 3.1 mV/N, which equates to a decrease of 22.5%.

After the fatigue tests, the mechanical structure of the piezoelectric sensor was investigated by micrograph analyses of the impacted point. Prior the analyses, the sensor was cut in a nitrogen bath in order to obtain a fragile break and a smooth cross-sectional surface.

**Figure 14:**
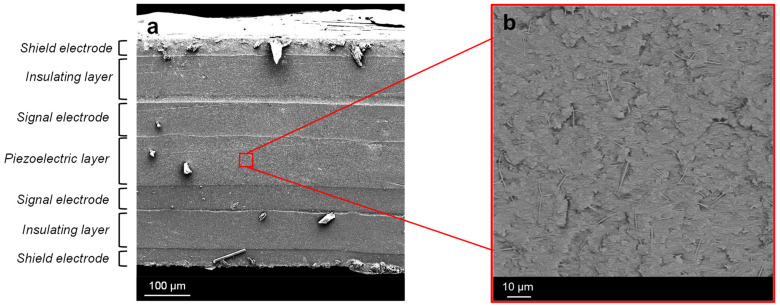
(**a**) SEM micrograph analyses of the piezoelectric sensor cross section (**b**) and of the nanofibrous layer embedded in the hosting matrix.

The SEM cross-sectional view of the multilayered piezoelectric sensor is shown in Figure 14a. The magnified midplane of the sensor in Figure 14b is the active part of the structure, where the embedding material (epoxy resin and polyurethane blend) permeated the highly porous PVDF-TrFE mat. It is worth highlighting that the use of nanofibers positively impacts on the mechanical performances of the composite material. Indeed, the use of a traditional PVDF-TrFE film could negatively affect the adhesion between the epoxy matrix and the fluorinated polymer. On the contrary, as is clearly observable in Figure 14a, no delamination between the nanofibrous midplane layer and the electrodes is observable, even after fatigue tests, as result of the intimate contact between the PVDF-TrFE nanofibers and the surrounding embedding matrix. Moreover, the use of CB-based electrodes resulted in a remarkable integrity of the sensor, which did not present delamination between the different layers. The use of traditional metallic layers could have compromised the adhesion with the polymeric matrix, in particular in cases of bending or high mechanical loads. Instead, the dispersion of CB nanoparticles in the polymeric matrix prevented any material discontinuities.

### 3.6. Tensile Strength Test

**Figure 15:**
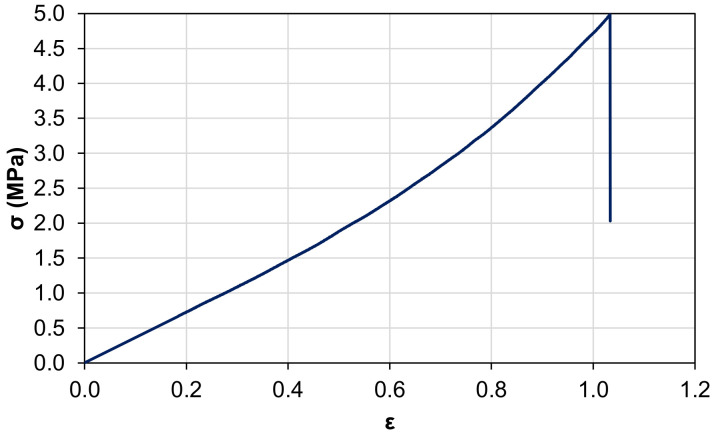
Stress vs. strain of the polymeric matrix of the developed sensor.

**Figure 16:**
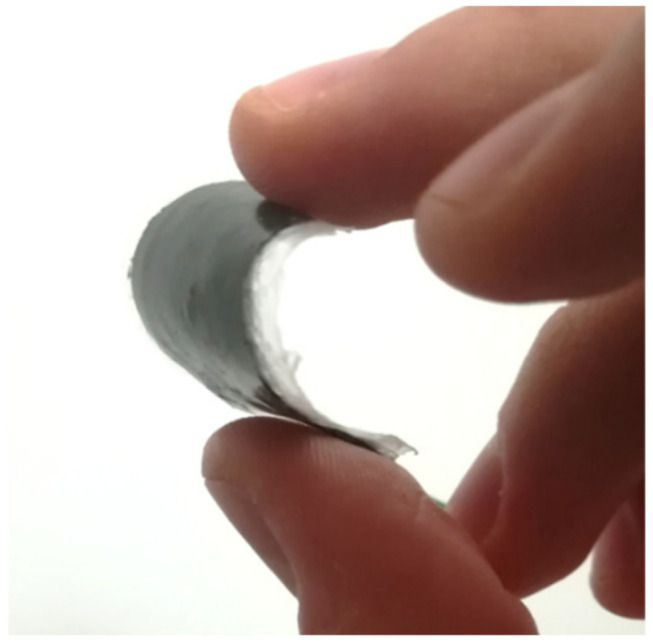
Image of the flexible piezoelectric sensor.

The specimen exhibits a stress trend typical of “elastic” materials, with a maximum elongation to failure of 104% and a maximum stress equal to 5 MPa, as shown in Figure 15. The elastic modulus is equal to 3.75 MPa, which is remarkably lower than those of standard epoxy resins, which range between 0.5 and 4 GPa. The final structure of the sensor is observable in Figure 16, where the black portion of the sensor surface corresponds to the electrode.

## 4. Conclusions

The flexible piezoelectric sensor proposed in this work was successfully fabricated by using PVDF-TrFE nanofibers. The piezoelectric nanofibers were embedded in a mixture of epoxy resin and polyurethane, and CB-based electrodes were added in a sandwich-like structure to avoid any materials discontinuities. The proposed hosting mixture—used both for nanofiber integration and electrode manufacturing—was tailored with the aim of producing an e-skin able to conjugate flexibility and mechanical resistance to high loads. The measured Young modulus and the micrograph analyses performed after the fatigue test demonstrated the effectiveness of the proposed sensor structure. The sensitivity of the sensor was measured in a frequency range typical of human activities, and the acquisition circuit parameters were accurately tuned in order to perform accurate detection of the applied force for each considered frequency value. The proposed polarization process remarkably increased the piezoelectric behavior of the nanofibers and represented an important step for the production of a highly sensitive flexible sensor (up to 4 mV/N). The linearity of the sensor was tested for compressive loads up to 1 kN, showing a coefficient of determination R^2^ equal to 0.997. The aforementioned characteristics of the fabricated sensor make it suitable for a wide range of fields, such as wearable applications or robotic fields (i.e., bonding onto robotic hands).
